# The double closet: Mental health needs of LGB college students from an intersectional perspective

**DOI:** 10.1371/journal.pone.0335710

**Published:** 2025-11-17

**Authors:** Athenai Ximena Sánchez-Millán, Marcela Tiburcio-Sainz, María Asunción Lara-Cantú, Tania Esmeralda Rocha-Sánchez

**Affiliations:** 1 Doctoral Program in Health Sciences, National Autonomous University of Mexico, Mexico City, Mexico; 2 Department of Social Sciences in Health, Directorate of Epidemiological and Psychosocial Research, Ramón de la Fuente Muñiz National Institute of Psychiatry, Mexico City, Mexico; 3 School of Psychology, National Autonomous University of Mexico, Mexico City, Mexico; 4 Department of Psychosocial Studies in Special Populations, Directorate of Epidemiological and Psychosocial Research, Ramón de la Fuente Muñiz National Institute of Psychiatry, Mexico City, Mexico; School of Nursing Sao Joao de Deus, Evora University, PORTUGAL

## Abstract

College is a crucial stage for personal development and can be particularly challenging for lesbian, gay, and bisexual (LGB) students as they face unique challenges related to their sexual orientation as well as social barriers. This qualitative study aimed to explore the mental health needs of LGB university students from an intersectional perspective. Twelve semi-structured interviews were conducted with seven bisexual, two homosexual, and three lesbian students at a public university in the state of Puebla. Content analysis with an intersectional perspective was used to explore their experiences to capture the factors affecting their mental health needs, from what leads them to seek mental health services to the moment they receive care and how that experience affects various areas of their lives. The findings suggest that LGB students often live in a “double closet,” concealing both their sexual orientation and their mental health, primarily from their families. From a university perspective, exploring this issue provides a framework for future interventions designed to address intersectional stigma in universities, through actions such as awareness-raising workshops and visual materials to create an inclusive environment and raise awareness about this issue and its consequences.

## Introduction

College students are subject to stressful experiences such as leaving the family, academic difficulties, financial problems, social pressures, alcohol consumption, pressure to achieve success, as well as problems related to sexual orientation and identity [1–3]. In this context, some young people use stimulants as a coping mechanism [4,5]. For example, it has been documented that methamphetamine and cocaine use among young people, including women and members of the LGB community, may be related to attempts to avoid traumatic experiences, social stigmatization, or the search for belonging and pleasure, particularly in environments where they face discrimination or violence [6]. In line with the above, approximately 65.2% of freshmen reported having experienced a mental disorder at some time in their lives, with 57.4% having done so in the past 12 months, particularly disorders such as anxiety and depression [7]. Furthermore, analyses show that the LGB population has a significantly higher risk of screening positive for ADHD (Attention Deficit Hyperactivity Disorder) (RR = 1.5) and problematic alcohol use (RR = 1.1). This is compounded by the additional stress that LGB individuals face due to their minority status. Minority stress refers to a conceptual model describing the stressors embedded in the social position of LGB individuals, which are perceived as causing health-related conditions such as mental disorders, psychological distress, physical disorders, health behaviors, and a general sense of poor well-being [8,9]. Stigma, prejudice, and discrimination create a hostile environment that exacerbates mental health problems in this population [9]. This stigma leads to inadequate responses and delays in seeking professional help, highlighting the urgent need to improve mental health literacy and reduce stigma in their immediate surroundings [10].

It is important to note that studies [11] such as the one exploring the issue of university students’ stigma towards mental health, found that levels of perceived stigma (M = 6.77, SD = 2.99) were significantly higher than those of personal stigma (M = 2.67, SD = 2.26), indicating a general overestimation of rejection on campus [10] (t (300) = –22.52, p < .001). Both types of stigma were also positively correlated (r = .312, p < .001), suggesting that those who perceive greater social rejection also internalize negative attitudes toward mental health.

However, to achieve a deeper understanding of the various facets affecting the mental health of LGB students, minority stress should not be considered the sole cause of emotional distress. Instead, what is required is a perspective enabling to understand the importance of the broader context in which LGB university youth exists. It was therefore decided to use intersectional analysis [12] to determine the factors affecting the mental health of university students who identify as LGB.

This methodological tool is part of feminist theory and “has enabled us to identify the diversity of interactions generated by very different types of subordination: for reasons of gender, sexual orientation, ethnicity, religion, national origin, (dis)ability, socioeconomic status and others” [13]. These include stigma, since this is a profoundly devaluing attribute, which degrades and demeans the person who bears it [14,15] and can be due to diverse attributes such as health, particularly mental health.

Health stigma arises when a person or group with a specific health condition experiences exclusion, blame, rejection, or devaluation. This is due to the anticipation or experience of a negative social judgment associated with that condition [15,16].

According to Meyer’s minority stress model, stigma affects LGB people at two levels: proximal and distal [9]. Proximal stigma refers to direct experiences of discrimination and rejection that occur in everyday interactions. This includes both interpersonal stigma, expressed through acts of discrimination by other individuals, and intrapersonal stigma, which arises from the internalization of negative messages and translates into self-stigma and self-devaluation. Conversely, structural stigma, rooted in discriminatory laws, policies and institutions, constitutes distal stress, a broader, more persistent factor.

The concept of “intersectional stigma” therefore describes how individuals or groups who experience multiple forms of discrimination due to their ethnicity, gender, or sexual orientation, face a convergence of stigmas that intensifies their negative effects [17]. It emphasizes [18] how the intersection of homonegativity, racism, and HIV (Human Immunodeficiency Virus) stigma affects access to services.

In consonance with the problems presented above, various studies show that LGB students experience greater problems with their mental health than their heterosexual peers [19–21].

The specific mental health challenges faced by the LGB community, such as societal discrimination, marginalization, and heteronormative expectations, are associated with significantly increased stress levels and higher rates of mental health problems compared to the heterosexual population [22]. Similarly, a cohort study in England found that lesbian and bisexual women, as well as bisexual men, experienced significantly less benefit from psychological therapy for depression and anxiety compared to heterosexual patients [21], revealing persistent inequities in care.

International literature has shown that the interaction between sexual orientation and other forms of marginalization, such as race/ethnicity or stigma associated with health conditions, intensifies the risk of mental health problems. A recent meta-analysis revealed that LGB adolescents and young people who have been victims of prejudice-based violence are 1.52 times more likely to experience suicidal ideation and 1.88 times more likely to have attempted suicide compared to their non-victimized peers [23]. In addition, family abuse was associated with a two-fold higher risk of suicidal ideation (OR = 2.01) and a 1.8-fold higher risk of attempting suicide. These findings underscore the need for targeted interventions to address the unique social stressors affecting this population, especially during adolescence and young adulthood, critical stages for the development of mental health. The COVID-19 pandemic (Coronavirus disease 2019) exacerbated existing mental health vulnerabilities among LGB youth, since isolation and the disruption of support networks had a significant impact, exacerbating stigma and discrimination [24,25].

Research has documented how multiple forms of oppression affect the daily psychological health of LGB people, creating fluctuations in their well-being. Negative experiences reinforce identity conflict and emotional distress, while positive experiences can improve mood [5]. Using a minority stress framework and intersectionality, Schmitz et al. qualitatively examined how young people conceptualize structural stigma due to the multiple structures affecting them (such as sexuality, gender, race/ethnicity, and age) and their mental health [25]. The findings highlight how Latinx LGB young adults experience structural racism, gender policing, and religious anti-LGB messaging in relation to their mental health. The study also reveals significant stigma toward depression within families and peer groups, which is problematic given its potential role in early detection and support. Despite growing international literature, few studies explore how these overlapping forms of stigma operate within Mexican public universities. Therefore, an intersectional approach is needed to understand how emotional needs emerge, are silenced, or transformed within the lived realities of LGB students.

In Mexico, major gaps persist in research on mental health disparities affecting university students who identify as part of the sexual diversity movement, despite legislative advances in LGB rights. Although international evidence has documented [22], increased risks of mood disorders, anxiety, and substance abuse in people with non-heterosexual sexual identities, most of these studies are conducted in developed countries, yielding little information on Latin American contexts and different cultural realities. In this respect, it is essential to generate empirical evidence from a local perspective that considers diversity within the spectrum of sexual orientations. Recent reports, such as the 2023 Situational Assessment of LGB People in Mexico, show that discrimination remains structural and multifaceted, highlighting the urgency of studies exploring how intersectional marginalization shapes mental health in university contexts. While recent studies underscore the need for intersectional approaches to understand how multiple systems of oppression shape the mental health of young people [22,24], much of this evidence originates from European or North American contexts, highlighting a knowledge gap in Latin American universities, where the impact of intersectional marginalization remains underexplored.

First-year university students in Mexico who identify as having non-normative sexual orientations have a higher prevalence of mental disorders and substance use, underscoring the need to design inclusive mental health strategies that acknowledge the multiple forms of vulnerability derived from stigma, discrimination, and the invisibility of sexual diversity [26]

Another study, [27] show that sexual minority students have a higher risk of suicidal ideation (ORs 2.05–3.00), suicide attempts (ORs 2.48–8.73), and non-suicidal self- harm (ORs 2.92–4.18) than their heterosexual counterparts, pointing to the need to study how factors such as perceived life stress mediate this relationship. On the other hand, [28] a study among college students referred a higher proportion of non-heterosexual individuals reporting anxiety compared to heterosexual students

In summary, the literature has documented increased risks of emotional disorders among LGB university students. However, there is still much to learn about how their mental health needs are influenced. This study, which adopts an intersectional perspective, aims to understand how various vulnerabilities impact their needs. This knowledge gap makes our work novel, as it qualitatively analyzes how stigmas related to sexual orientation and mental health intersect, shaping what we call a “double closet.” Furthermore, by focusing on a Mexican public university, it provides localized evidence on a phenomenon that has been underdocumented in Latin America.The aim is to answer the question: What are the mental health needs of LGB university students from an intersectional perspective?

This article contributes to the literature by introducing the concept of the “double closet” in the context of Mexican higher education, revealing how the need to conceal both one’s sexual identity and mental distress creates a compounding effect on emotional suffering. By analyzing these dynamics through qualitative testimonies, this study expands current understandings of intersectional stigma in LGB populations. In this research, we chose to focus the analysis on university students who identify as lesbian, gay, or bisexual (LGB), the decision was made mainly on methodological issues, on the one hand, the availability of participants and, on the other hand, the need to generate more robust evidence regarding the mental health of LGB students in Mexico, who constitute one of the most highly represented groups and for whom there is prior evidence of specific vulnerabilities. However, we recognize the diversity of orientation as part of the LGBT+ spectrum as well as the relevance of including all identities, orientations, and expressions.

## Method

A qualitative exploratory study was conducted to achieve the aim. This approach allows for a deeper understanding of understudied phenomena, such as the mental health needs of the LGB university population, from an intersectional perspective [29]. Category analysis was used to organize and classify the qualitative data, while the intersectional framework revealed the various forms of oppression and discrimination experienced by LGB students [30].

### Participants

The goal was to select a sample of LGB university students from a public university in the city of Puebla. The number of participants was determined by data saturation [31], a criterion used in qualitative studies to continue data collection until no further new information emerges. The inclusion criteria were as follows: being Mexican, aged between 18 and 25 years old, identifying as LGB, and enrolled in the university. Those who chose to have the interview online also had to have access to the internet and an electronic device.

### Instrument

To explore the mental health care needs of LGB college students, a semi- structured interview was used as a data collection method. First, a pilot test was conducted to explore whether the questions were sufficiently clear and relevant to obtain the necessary information. Conducting a semi-structured interview allowed flexibility in the sequencing and formulation of questions, ensuring that the responses accurately reflected participants’ experiences [32]. Moreover, the structuring of the interview provided legitimacy and trust in the process, without imposing absolute control over the interviewees [33].

The interview guide comprises 21 questions grouped into the following categories: introduction and informed consent, rapport and personal context, mental health experiences, sexual orientation and identity, minority stress, mental health care needs, mental health and coping, and closing the interview. The topics included in the interview guide are based on minority stress theory [9] and the intersectional perspective [34]. These theories allowed for the structuring of categories related to mental health experiences, the impact of social stigma, family support or rejection, coping strategies, and perceptions of the university environment.

The interview guide allowed for a comprehensive exploration of the mental health needs of LGB university students, considering how they are shaped by the intersection of sexual orientation, social stigma, and structural conditions. This instrument facilitated the collection of relevant qualitative data to understand how intersectional stigmas influence the experience, expression, and treatment of emotional distress in this population.

### Procedure

An invitation was sent through Facebook and posters posted in psychological clinics. The inclusion criteria were detailed in the invitation to ensure that those interested in participating met the study requirements; eligible LGB students contacted the principal investigator by email. The interviews were conducted by the lead researcher, a cisgender bisexual woman, with prior experience as an interviewer in qualitative studies. For this project, she received specific training from the co-authors to ensure a sensitive and inclusive approach aligned with the research topic. No prior relationship existed between participants and the research team; individuals responded to an open call and made first contact to express interest. Participants had no previous knowledge about the interviewer. All interviews were individual, and only the interviewer and the participant were present. Fieldwork was conducted during the COVID-19 pandemic, from March to November 2022, adapting data collection to two modalities: online and in-person. In-person interviews were conducted in an office located on the university premises, while the online interviews were conducted using the Zoom platform. No participant declined to participate or withdrew from the study. Prior to each interview, participants were read an informed consent form and were reminded of their right to withdraw at any time, though none exercised this option. No repeat interviews were conducted, and no field notes were taken, partly due to the logistical constraints imposed by the COVID-19 pandemic. Interviews lasted approximately one hour. Transcripts were not returned to participants for review or correction.

### Data analysis

A category analysis was performed to organize and understand the qualitative data [29]. The process included the following stages: All the interviews were transcribed and coded, assigning labels and meaning to each text fragment [35]. The conceptual frameworks of intersectionality and minority stress were used to organize the information into categories and subcategories. These categories represent units of information that enabled us to understand the participants’ experiences [36,37]. The “meaning categorization” technique [38] was used to analyze the data. Patterns and relationships between the categories were identified, giving meaning to the participants’ discourse [24]. To analyze the data, patterns and relationships among the categories were identified, providing insight into the participants’ responses. An iterative process of reading and rereading the transcripts was employed. Additionally, an a priori list of categories was created and refined throughout the analysis.

### Ethical considerations

This study was conducted in accordance with the Department of Health, Education and Welfare ethical principles of human research [34,38], considering privacy, data security, confidentiality and handling of sensitive information, information accuracy, participant experience, technological literacy and accessibility [39]. Informed consent and procedures were approved by the Research Ethics Committee (REC) of the university where the study was conducted (SIEP05/2022).

### Characteristics of participants

The sample comprised 12 university students, with an average age of 20.75 years (SD = 0.39). In regard to gender identity, three of the participants identified as cisgender men (25%), one as a transgender man (8.3%), and eight as cisgender women (66.7%). In regard to sexual orientation, seven participants identified as bisexual (58.3%), three as lesbians (25%) and two as homosexuals (16.7%) (Table 1)

**Table 1:** Characteristics of participating students (N = 12).

Pseudonym	Age	Sexual orientation	Gender identity
Edna	20	Lesbian	Cisgender woman
Carmen	19	Bisexual	Cisgender woman
Eloisa	22	Bisexual	Cisgender woman
Lana	22	Lesbian	Cisgender woman
Mauricio	19	Bisexual	Cisgender man
Juan	20	Bisexual	Transgender man
Claudia	22	Lesbian	Cisgender woman
Pedro	22	Homosexual	Cisgender man
Tadeo	19	Homosexual	Cisgender man
Jazmín	20	Bisexual	Cisgender woman
Gabriela	22	Bisexual	Cisgender woman
Pamela	22	Bisexual	Cisgender woman

Thematic analysis based on the semi-structured interviews yielded seven main categories enabling us to understand the experiences and needs of LGB university students in relation to their mental health. Each category is divided into subcategories that contributed specific elements illustrated with representative quotes from the participants. The following table summarizes these categories, subcategories and relevant examples (Table 2).

**Table 2:** Categories, subcategories and relevant examples.

Category	Subcategory	Example
Trajectory of recognition and stigma		
1. Acknowledgment of emotional needs in contexts of invisibility	Self-exploration without references	*“In fact, the subject, as well as homosexuality, was very foreign to me, because then Istarted to think about it and I think I wasn’t really aware of it”* (Gaby, 20 years old)
	Confusion and emotional insecurity	*“When I was little, like in kindergarten, at the beginning of elementary school, I remember there was a girl, you know? Well, I loved her very much (laughs) but at one point I remember very clearly because it is burned into my memory and I wondered, ‘Is it love or is it friendship?’ I questioned something like that. Besides, I went to a Catholic school, a religious one.”* (Edna, 20 years old)
	Need for information as the basis for self-care	*“I remember in a movie it was like, ‘Oh, okay, I get it!’ I remember it was when I saw that movie that I said to myself, ‘Okay, this is normal, this exists. Marta Higareda was in it, I think, when she was younger.”* (Edna, 20 years old)
2. Internalized stigma: from identity rejection to rejection of emotional self- care	Feelings of abnormality	*“I felt strange things, and I said, ‘This is wrong’ to one of my classmates, and ‘This is wrong and this looks bad,’ because that was the idea I had that this was wrong, that it wasn’t right.”* (Carmen, 19 years old)
	Rejection of one’s own identity	*“I didn’t accept myself, I didn’t have, in fact, I keep asking myself why me? Why not someone else? Why not the neighbor? Why not, a guy 30 km away or from Atlixco? Why me?” And that’s a question I’ve asked myself many times. “I tell you, I keep asking myself that question, and it’s hard.” (Pedro, 22 years old)*
Double closet and familial and institutional barriers		
3. Double closet: hiding orientation and discomfort	Family reactions to coming out	*“At first it was a little bit… they reacted badly… then well, they told me how great it was that I had accepted myself and so on.” (*Edna, 20 years old)
	Minimization or pathologization of suffering	*“When I told them I was gay, my parents said: “Time to go to the psychologist.””* (Tadeo, 19 years old)
4. Stigma towards mental health as a barrier to access	Stigma towards therapy and psychiatry	*“At first, it was like, I had the idea that the psychiatrist was for when you’re seriously ill... but I realized that’s not what it’s for. You can go for other things that don’t necessarily mean you’re in really bad shape.” (Jazmín, 20 years old)*
	Experiences of discrimination in services	*“We went to a normal psychologist, but then, um, as they didn’t see that I was making progress to going back to being heterosexual according to them (parents), you know, so they sent me to a priest who had a degree in psychology... because it was like, I told my dad, you know, I don’t want to see him anymore, I feel very uncomfortable talking to him, because literally I feel very judged.”* (Tadeo, 19 years old)
	Fear of being judged by others	*“Yes, absolutely (speaking of her family’s prejudices about the subject of mental health), I buy and hide the medication that the psychiatrist prescribes for me.”* (Lana, 22 years old)
Structural conditions		
5. Structural factors that aggravate mental health needs	Academic stress and performance pressure	*“…the stress is mostly linked to class assignments, that’s what has affected me most.”* (Edna, 20 years old)
	Financial instability	*“It stresses me out… just not having money.”* (Lana, 22 years old)
	Isolation due to outsider status	*“I’m from out of town, so I feel like it was hard for me to adapt to this whole process of living alone, the degree course, it was kind of hard.”* (Claudia, 22 years old)
Moments of help-seeking and re-signification		
6. The search for support as a response to accumulated discomfort	Crisis as a turning point	*“Yes, one, yes, like the main reason I went, it was one of those events and I said to myself ‘Oh, ¡I can’t take it anymore!’ that’s why I sought help...” (*Pamela, 22 years old)
	Academic, emotional or identity motivation	*“My parents had found me a therapist by the time the pandemic started…”* (Mauricio, 19 years old)
	Awareness of the need for psychological help	*“...my mom also changed her attitude when she saw that in our family we started talking more about therapy…”* (Jazmin, 20 years old)
7. Resignification of mental health as a right and self-care	Recognition of therapy as a valid tool	*“I think that if you need help, accompaniment is excellent. Often you need it and you don’t go, but taking care of your mental health is just as important as taking care of your physical health. I feel like we should all do it.”* (Claudia, 22 years old)
	Change in family attitude towards psychological care	*“...my mom also changed her attitude when she saw that in our family we started talking more about therapy…” “She no longer sees it as something bad, but as something normal.”* (Jazmín, 20 years old)

### Recognition of emotional needs in contexts of invisibility

This category sought to explain how discomfort begins to be felt, from a combination of emotion and confusion, coupled with disinformation.

#### Self-exploration without references.

The following testimonial clearly reflects how the lack of information on sexual diversity in childhood and in religious school settings creates confusion. Edna, 20, recounted her process of self-discovery in a religious school environment where sexual diversity was not discussed:

*“So they never talked to us about that, there was no information about diversity, about what you might like and so on, until the day I asked myself why am I feeling this? What is this?” (Edna, 20 years old)*

Gaby, 20, mentioned that homosexuality was a subject of which she was barely aware, which made it difficult for her to recognize herself:

*“In fact, the subject, like homosexuality, was very foreign to me, because then I started to think about it and I think I wasn’t really aware of it.” (Gaby, 20 years old)*

Both Gaby and Edna explain how this structural ignorance delayed acknowledgement of their sexual orientation. The search for meaning, often mediated by external cultural references such as cinema, reveals a malaise linked not to an “individual condition,” but to a structural social omission.

#### Confusion and emotional insecurity.

This section shows the moments where, due to the failure to understand what they are feeling, people experience bewilderment, ambiguity, and emotional doubt.

*“When I was little, like in kindergarten, at the beginning of elementary school, I remember there was a girl, you know? Well, I loved her very much (laughs) but at one point I remember very clearly because it is burned into my memory and I asked myself, ‘Is it love or is it friendship?’ I asked myself something like that. Besides, I went to a Catholic school, a religious one.” (Edna, 20 years old)*

*“It was like I subconsciously knew it existed, but I didn’t think about it.” (Gaby, 20 years old)*

These experiences reflect an unnamed emotional need, where the lack of role models and information about sexual diversity causes emotional insecurity, making it difficult to recognize discomfort early.

#### Need for information as the basis for self-care.

This section shows how exposure to external information (such as the cinema, media or culture) becomes the first resource for recognizing and validating orientation.

*“I remember in one movie it was like, ‘Oh, okay, I get it!’ I remember it was when I saw that movie that I said to myself, ‘Okay, this is normal, this exists. Marta Higareda was in it, I think, when she was younger.’” (Edna, 20 years old)*

Here, Edna is showing that what the educational environment did not give her (information and validation), she found symbolically in a movie. That scene constituted the first step towards “this is normal.”

In short, the testimonials show that the lack of accessible information and references about sexual diversity during childhood and adolescence creates confusion, making it difficult to understand what one is feeling. External information, when it appears, can become an initial resource for emotional validation.

#### Internalized stigma: from identity rejection to the rejection of emotional self- care.

The following approach seeks to show how internalized stigma affects identity and acceptance. It was considered essential to include this section because it shows that a mental health need is not always expressed as a visible symptom but can be rooted in subjective processes such as shame about being who one is, guilt about feeling different, or the feeling of not deserving help.

#### Feelings of abnormality.

Participants’ accounts describe how the internalization of social norms that prioritize heterosexuality and devalue other sexual orientations affects their self-perception. Carmen, 19, expressed feelings of “abnormality” when experiencing the following:

*“I felt strange things, and I said. ‘This is wrong’ to one of my classmates, and ‘This is wrong’ and ‘This looks bad,’ because that was the idea I had that this was wrong, that it wasn’t right’ (Carmen, 19 years old)*

*“I didn’t feel like it was okay for me to like men, because well, because of the education I had received, I was like, I was tormenting myself all the time saying, no, it can’t be like that, because what are you going to tell your mom?” (Pedro, 22 years old)*

#### Rejection of one’s own identity.

The following testimonials describe the experience of being unable to accept oneself, of wishing not to be who one is, or of rejecting oneself.

*“I felt like a monster back then because of my ignorance and everything they told me at home, it seemed like I didn’t fit in” (Carmen, 19 years old)*

*“I didn’t accept myself, I didn’t have, in fact, I keep asking myself why me? Why not someone else? Why not the neighbor? Why not a guy 30 km away or from Atlixco? Why me?” And that’s a question I’ve asked myself many times. “I tell you, I keep asking myself that question, and it’s hard.” (Pedro, 22)*

These stories show how internalizing the stigma surrounding sexual orientation can translate into a profound rejection of one’s own identity, generating feelings of shame, guilt, and self- effacement. Difficulty accepting oneself does not arise in isolation, but rather as a result of family and social environments that systematically invalidate LGB identities.

### Double closet: concealing orientation and discomfort

This section, which refers to the “double closet,” refers to when LGB college students not only conceal their sexual orientation or gender identity for fear of social rejection but also conceal their mental health needs due to the associated stigma.

#### Family reactions to coming out.

Family reactions to the revelation of sexual orientation act as a thermometer for structural heteronormativity. The following testimonials show how their families reacted when participants decided to reveal their sexual orientation. The experiences reflect a range of family responses ranging from acceptance to denial.

Edna, 20, shared her experience:

*“At first it was a little bit… they reacted badly… then they reacted well, they told me how great it was that I had accepted myself and so on.” (Edna, 20 years old)*

Jasmine, 20, described how her mother’s attitude changed after she revealed her sexual orientation:

*“…she didn’t say anything to me, but her attitude towards me changed a little bit, so I felt like she wanted to tell me something, but she didn’t know how to, or for example, when we were watching something on television about, um, the community, she would get serious or she would just look at me for a while, and I was like, Oh! But she never said anything, until one day she just told me, I think it was before I came out, she said, ‘Come’ and I went, and she said, she started telling me that she loved me, that I was her daughter, that she would always love me no matter who I was, so I felt that that was what she meant.” (Jazmín, 20 years old)*

At the same time, there is another closet, the “second closet.” A key finding in the testimonials is the perception of mental health as a “closet” parallel to that of sexual orientation. The fear of being judged socially for seeking psychiatric help creates a process similar to hiding one’s sexual identity, which reinforces intersectional stigma in LGB communities.

*“At first it was like, I through that the psychiatrist was like for when you are seriously ill, or when you are older, I don’t know, because in my experience my grandparents would get sick and would wander the streets. So it was like, that was the expectation, of ah, well, when a person is like that, they already need a psychiatrist, but well it’s like, no, well I saw that it’s not for that, you can go for, for other things that don’t necessarily mean that you are in really bad shape. So, it was a bit hard for me to understand it, how it worked.” (Jazmín, 20 years old)*

*“Well, at first it was very difficult for me. I didn’t tell anyone and I was very ashamed and so on. Now I feel like it says a lot about a person’s maturity to understand that it’s necessary, that sometimes you need to go somewhere and cry and look for all these tools that help you get through difficult emotional times. So I feel like my idea of going to the psychologist was linked to a place for crazy people when it’s actually for healthy people.*

*With the psychiatrist, well, there it’s a little different. I still try to have the same opinion as I did with the psychologist, but it’s still very difficult for me to accept that I’m not crazy. It’s still been a difficult thing to cope with, yes, I mean, this is the same as being gay, right? I mean, outwardly I justify it, but when it comes to me, I don’t. So I feel that, if you talk about a person who is not well, I don’t know if in a sense... I don’t know, that they are not stable, that they are not normal, that they are not well, that they are sick... (laughs) ah, yes, yes it is something that is much more difficult for me to tell people, it scares me, I feel that they could react differently... I think that would be an accurate way to describe it, there is a closet around seeing a psychiatrist.” (Lana, 22 years old)*

This testimonial demonstrates the anticipation of rejection, which generates emotional hypervigilance that prevents timely access to help. Like the sexual orientation closet, the mental health closet is marked by fear of discrimination and external judgment.

#### Minimization or pathologization of suffering.

Family stigma operates by pathologizing homosexuality and minimizing emotional pain. Tadeo, 19, shared the following:

*“When I told them I was gay, my parents said, ‘Time to go to the psychologist.’” (Tadeo, 19 years old)*

This response is not an isolated event but can be understood as part of a heteronormative logic that reinforces the idea that LGB identities should be corrected or treated clinically. The minimization of emotional pain by family figures illustrates adult-centrism [32], a system that invisibilizes the struggles of young people, especially women and queer people, under the premise that youth is equal to lack of experience.

Carmen, 19, shared this account:

*“...but I had several traumas in my childhood, that too, like my mom didn’t want to notice and neither did my family, like they ignored it, but I did seek a lot of help, because I think what I felt wasn’t normal, I mean I didn’t really want to get up, when I was a primary or secondary school student, I mean, like I didn’t see that as very normal [...] Now, in fact, there was a point when I had a crisis, when I said, “Enough is enough! I can’t take it anymore! I need peace! And since I studied graphic design and I handle cutters and scissors, I was about to stick them into my neck and I said, ‘No! Why are you going to do that? It got to the point where I said, ‘You need help!’ Because I still want to get ahead.” (Carmen, 19)*

This story underscores the urgency of building environments where no one should have reached their limit before having access to health services.

The above stories show that the family environment can deny emotional distress and pathologize a different sexual orientation.

#### Structural factors that exacerbate mental health needs.

The mental health needs of LGB college students cannot be understood in isolation from the structural conditions that shape their daily lives. This section brings together three key dimensions: academic stress and performance pressure, economic instability, and the isolation that comes with being from out of town, to show how these conditions impact LGB students. Analyzing these experiences from an intersectional perspective shows that sexual orientation and gender identity intersect with other axes of inequality such as class, age, place of origin, and relationships with authority figures.

#### Academic stress and performance pressure.

The university academic environment constitutes a significant source of stress for students. Academic stress manifests itself not only in the workload, assessments, or presentations, but also in the way one experiences observation, judgment, and expectations on the part of authority figures.

*“Being under pressure, like from an academic, like, like, presentations, especially when someone, like, higher-ranking, so to speak, is watching me or wants me to express my ideas or something like that, it gives me, makes me very anxious...” (Jazmín, 20 years old)*

*“I know I have to do that, but I’m too lazy so I don’t do it, and then well, it’s a vicious cycle, because I also get stressed because I don’t do it, but I don’t feel like doing it...” (Pedro, 22 years old)*

*“…the stress is mostly due to class assignments, that’s what has affected me the most.” (Edna, 20 years old)*

These testimonials show that academic stress, far from being a neutral experience, becomes more complex when combined with the experience of being an LGB person. Test anxiety, procrastination, and constant pressure to perform reveal the need to create university spaces that are more sensitive to the impact of stress. Including this analysis in the discussion on mental health makes it possible to see that the needs of LGB students are not limited to their sexual orientation but rather emerge at the intersection with other structural factors.

#### Financial instability.

Financial instability represents a key structural factor that directly impacts the emotional well-being of university students, and its effect is exacerbated when it combines with experiences of discrimination based on sexual orientation and restrictive family dynamics. In contexts where financial support networks are limited or conditioned by homophobic and sexist attitudes, LGB people may face constant stress related to financial uncertainty, difficulty paying for their studies, and the need to prioritize survival over emotional self-care. This intersection of economic precariousness and sexual stigma reveals how material conditions also operate as barriers to mental health.

*“It stresses me out… just not having money.” (Lana, 22 years old)*

*“I say that more than the financial situation, like we used to rent and now we continue renting, but before it was three incomes and now it’s only two... we stopped doing those things, more than anything to be punctual and so that I can be more punctual at the University with my work.” (Carmen, 20 years old)*

*“Mm yes, and it’s also linked to financial things, because there’s my allowance, so I feel like he’s capable of taking it away from me. His machismo and homophobia are so intense that it’s very likely that he’ll say, ‘You no longer exist for me... I’m not going to give you my financial support either’…” (Carmen, 20 years old)*

The above stories show that a lack of financial resources not only limits access to basic needs and support services, but also creates persistent emotional distress, feelings of instability, and additional pressure to meet academic demands despite adversity. For LGB students, economic dependence on family figures who spread homophobic or sexist rhetoric can become a source of blackmail and control, further weakening emotional autonomy. Addressing mental health from an intersectional perspective therefore requires recognizing that structural conditions, such as poverty or economic inequality, are not neutral: they affect those who already experience other forms of marginalization differently and must be considered in any policy or intervention designed to enhance the well-being of LGB university students.

#### Isolation due to being an out-of-town student.

Being a out-of-town student involves undergoing an adaptation process that goes beyond geographical change. For the participants in this study, this transition often also involves distancing themselves from support networks, rebuilding their sense of belonging, and confronting social or family norms that can create tension. Physical isolation from family, the need to manage daily life independently, and the forced return home during the pandemic are experiences which, taken together, significantly affect mental health. Discussing this phenomenon is essential for intersectional research, as it sheds light on how student mobility intersects with identity and structural factors that complicate emotional well-being.

*“My only complaint is that I’m not with my parents like I was before, in fact, I’m hardly ever with them, but obviously I miss my family like that.” (Mauricio, 19 years old)*

*“I’m from out of town, so I feel like it was hard for me to adapt to this whole process of living alone, my degree, it was kind of hard.” (Claudia, 22 years old)*

*“I think that was the hardest part for me. I mean, it was like, well, awful, you know? Because apart from the independence… more than anything, you have your own space and your own schedule, so I feel like it’s hard to adapt to your family’s schedule.” (Jazmín, 20 years old)*

Testimonials show that emotional uprooting, loneliness, and difficulties readjusting to family life are key factors that shape psychological distress in out-of-town students, especially when there are gender or sexual tensions within the family. In contexts where independence represents a space of freedom for the expression of identity, returning to controlling environments can exacerbate stress and emotional vigilance. Incorporating an analysis of isolation due to being an out-of-town student into the study of the mental health needs of LGB students allows for a broader understanding of the factors that influence their vulnerability and reinforces the urgent need to create support policies recognizing the intersection of mobility, identity, and emotional care.

The stories analyzed in this section show that structural conditions such as academic, economic, and geographic factors exacerbate performance stress when combined with fear of judgment or exclusion. Financial insecurity not only reflects material shortages but also a scenario of blackmail and identity control; and the experience of being an out-of-town student can accentuate loneliness, exposure to oppressive family dynamics, and difficulty accessing safe spaces. Discussing these factors is therefore not a diversion from the central objective of this research, but rather a way of delving deeper into it. Recognizing how social structures interact with diverse identities allows for a broader understanding of the mental health needs of LGB college students.

#### Help-seeking as a response to accumulated discomfort.

The search for psychological support among LGB university students does not arise in a vacuum, but as a response to the accumulation of discomfort that develops over time in contexts marked by academic demands, structural violence, discrimination, and emotional exclusion. The following stories show how, faced with emotional exhaustion, existential crises, or the tiredness caused by stigma, many people turn to therapy for urgent or preventative care. It is essential to address this when the goal of the study is to identify the mental health needs of this population, as it helps us understand that the demand for psychological care not only responds to individual symptoms but is intertwined with structural conditions and intersectional experiences.

#### Crisis as a turning point.

Within the emotional journey of LGB university students, psychological crises often become turning points that mark the beginning of the search for professional help. These crises do not arise in isolation, but are the result of the accumulation of structural, emotional, and social factors that permeate their lives. This subcategory highlights how crises, whether a breakup, a bout of hopelessness, or suicide risk, act as triggers for seeking help, underscoring the urgent need to address distress before it becomes unbearable.

*“Yes, one, yes, like the main reason I went, it was one of those events and I said to myself ‘Oh, I can’t take it anymore!’ that’s why I sought help... (Pamela, 22 years old)*

*“...I said, ‘Enough is enough, I can’t take it anymore, I need peace... I was about to stick them in my neck and said, ‘No! What’s the point of that?’ I got to the point where I said, ‘You need help.’” (Carmen, 19 years old)*

*“...my attitude had changed radically, so I decided to ask for help. I went to the College Administrative Building, told them what had happened, and then they referred me.” (Juan, 20 years old)*

The stories of Pamela, Carmen, and Juan show that, for many LGB people, access to psychological care is not preventative, but rather reactive, when distress has already overwhelmed their personal resources. The experiences described reflect the discomfort of feeling emotionally exhausted, alone, or helpless, and how, in these extreme moments, psychological intervention appears as a possibility of protection.

#### Academic, emotional or identity motivation.

Many LGB university students not only seek psychological support because they are experiencing a visible or acute crisis, but because they feel continuous distress that affects their academic performance, emotional stability, or identity process. This category allows us to observe how becoming discouraged about one’s degree course, the impact of previous traumatic experiences, and the pandemic context acted as catalysts that triggered the decision to seek help. These stories show that, for many people, access to mental health care is not only activated in extreme moments, but also as a support strategy for understanding, sustaining, and resist the everyday emotional strain.

*“I took a workshop… at the BUAP on anxiety and because of the pandemic… you don’t feel the same way about your degree course anymore…” (Edna, 20 years old)*

*“Yes, I think it’s more discouraging right now, because you don’t feel the same way about the course anymore, it’s been like three semesters… you get discouraged…” (Edna, 20 years old)*

*“When I was a child, I was sexually abused… as I talked about it, it was hard… then I found out that ARPA was offering an art therapy workshop and I said, ‘OK, this is my chance.’” (Pamela, 22 years old)*

*“My parents had found me a therapist by the time the pandemic started…” (Mauricio, 19 years old)*

These experiences show that emotional well-being cannot be understood solely through clinical indicators or emergencies, but also through factors that slowly damage mood, personal safety, or the sense of belonging. Incorporating this dimension into the analysis highlights the value of preventive spaces, such as workshops and timely support, and underscores the importance of university policies that recognize these motivations as legitimate and urgent. From an intersectional perspective, acknowledging these forms of distress allows us to care for those who experience multiple vulnerabilities and move toward truly inclusive mental health.

#### Awareness of the need for psychological help.

Recognizing the need for psychological support constitutes an act of agency and care that, in the case of LGB university students, takes on special relevance due to the historical stigma associated with both sexual diversity and mental health. This subcategory includes testimonials that show how some people, after periods of prolonged emotional suffering, come to realize that they need professional help as a fundamental step toward their well- being. This awareness-raising process not only reflects the search for relief from discomfort but also constitutes a generational shift in the way emotional care is understood and valued, even within families that previously dismissed the importance of therapy.

*“I think more than anything, not feeling that mental burden anymore… I was crying all the time… I didn’t feel very well anymore…” (Carmen, 19 years old)*

*“...now I feel that it says a lot about a person’s maturity to understand that it’s necessary, that sometimes you need to go somewhere and cry…” (Lana, 22 years old)*

*“...my mom also changed her attitude when she saw that in our family we started talking more about therapy…” (Jazmín, 20 years old)*

*“...I asked my mom ‘Why don’t you go to therapy?’ and she said, ‘I don’t need it’... but in my case, when I told her, she supported me... (Pamela, 22 years old)*

Discussing the need for psychological help is essential in research on mental health from an intersectional perspective, as it sheds light on how LGB students transition from silence or self-imposed demands to openness to emotional care. This awareness, in contexts where prejudices about “being weak” or “being wrong” for asking for help still persist, is a political act of resistance and self-affirmation. Recognizing these processes allows us to broaden our focus beyond acute crises and to assess the role that awareness, emotional education, and support networks play in the decision to seek therapeutic support.

The ways LGB college students choose to seek psychological support allow us to understand that mental health cannot be addressed from an individualistic or decontextualized perspective. The experiences described show that the demand for care does not always occur in a planned manner or within a preventative model, but rather, in many cases, is the result of emotional saturation due to accumulated experiences of violence, exclusion, or vulnerability. At other times, it arises from a reflective, academic or identity-based motivation that expresses the desire to understand oneself better and to find a safe space to inhabit one’s experience. Visibilizing these access routes to help not only enables one to map the factors that impact the psychological well-being of LGB students, but also to question institutional models that assume that all people access care on equal terms.

### Stigma towards mental health as a barrier to access

Addressing stigma as a structural barrier to accessing mental health care is essential when attempting to understand the needs of LGB college students from a holistic perspective. This stigma takes various forms, from prejudice toward therapy and psychiatry to experiences of discrimination within care services themselves that discourage people from seeking support. The testimonials analyzed in this section show how the double burden of stigmatization— due to sexual orientation and emotional needs—increases psychological distress, restricts the agency of those trying to take care of their mental health, and reinforces isolation. Making these obstacles visible is essential for thinking about more inclusive care policies and therapeutic models that recognize the intersection between identity and emotional suffering

#### Stigma toward therapy and psychiatry.

Despite advances in raising awareness about mental health, the stigma surrounding psychotherapy and psychiatry continues to influence decisions about seeking help. This subcategory shows how many people still associate psychological or psychiatric care with ideas of “madness,” “weakness,” or “abnormality,” which hinders early, voluntary access to support services. In contexts where the stigma surrounding sexual orientation already conditions emotional expression, the fear of being labeled “unbalanced” or “sick” reinforces silence and the postponement of care. The narratives gathered here demonstrate how prejudices about mental health are intertwined with experiences of discrimination and structures that invalidate psychological suffering, creating additional barriers to well-being.

*“Well, at first it was very difficult for me. I didn’t tell anyone and it makes me feel very sorry and all that. Now I feel like it says a lot about a person’s maturity to understand that it’s necessary, that sometimes you need to go somewhere and cry and seek all these tools precisely that help you get through difficult emotional moments. So I feel like my idea of going to the psychologist was linked to a place for crazy people, whereas in fact it’s fo healthy people. With the psychiatrist, well, there it’s a little different. I still try to have the same opinion as with the psychologist, but it’s still very difficult for me to accept that I’m not crazy. It’s still been a difficult thing to cope with, yes, I mean, this is the same as being gay, right? I mean, outwardly I justify it, but when it comes to me, I don’t. So I feel that it refers to a person who is not well, I don’t know if in a sense... I don’t know, that they are not stable, that they are not normal, that they are not well, that they are sick... (Lana, 22 years old)*

*“At first, it was like, I had the idea that the psychiatrist was for when you’re seriously ill... but I realized that’s not what it’s for. You can go for other things that don’t necessarily mean you’re in really bad shape.” (Jazmín, 20 years old)*

Opening the conversation about the stigma surrounding therapy and psychiatry is critical when exploring the mental health needs of LGB college students, as they act as silent but persistent barriers to accessing timely care. Realizing that the idea of “being unwell” still carries a negative symbolic weight, highlights the urgency of transforming the social and family narratives that pathologize both sexual diversity and the search for emotional help.

### Experiences of discrimination in services

One of the most significant barriers to effective access to mental health care for LGB college students is discrimination within therapeutic care itself. The experiences shared by participants show that, far from being safe, affirming spaces, some psychological care settings reproduce stigmas regarding sexual orientation and gender identity. This type of discrimination, whether explicit, such as refusing to recognize a person’s pronouns, or implicit, such as pathologizing their emotions or identities, not only prevents the development of an effective therapeutic relationship but also exacerbates the psychological distress that prompted the person to seek help. Analyzing these experiences sheds light on why the LGB university population faces greater obstacles to taking care of their mental health.

*“We went to a normal psychologist, and, but then, um, as they didn’t see that I was making progress to going back to being heterosexual (my parents), you know, they sent me to a priest who had a degree in psychology... because it was like, I told my dad, you know, I don’t want to see him anymore, I feel very uncomfortable talking to him, because I feel very judged.” (Tadeo, 19 years old)*

*“Well, once I went to see a psychologist about my identity, who didn’t want to address me with my pronouns, and he thought that I was somehow wrong, and that’s why I stopped going.” (Juan, 20 years old)*

*“I don’t remember much, it was like she was weird. I mean, I would tell her how I felt and all that, and she... but I felt like she was implying that what I felt was wrong, that I shouldn’t feel that way, etc. So, well, I don’t know, I didn’t feel comfortable, I didn’t feel confident, I felt like she was judging me all the time...” (Eloísa, 22 years old)*

The testimonials of Tadeo, Juan, and Eloísa show that access to mental health care is not limited to the availability of services, and instead depends on their quality, safety, and sensitivity to sexual and gender diversity. These experiences highlight the urgent need to train mental health professionals who are not only free from prejudice but are also equipped with the ethical and clinical tools to accompany students whose identities have been historically marginalized, with respect, competence and validation.

#### Fear of external judgment.

Fear of external judgment is an invisible but profoundly influential barrier to seeking and continuing mental health care.In the case of LGB university students, this fear is not only related to the widespread social stigmatization of mental disorders but also intersects with previous experiences of rejection based on sexual orientation or gender identity. The following narratives reveal how the perception of being judged or delegitimized by family figures, especially in contexts where adult-centrism or ignorance predominates, creates a need for concealment, inhibits emotional expression, and hinders free and sustained access to psychological and psychiatric services. Analyzing these types of experiences is key to understanding the multiple layers of invisibility and surveillance affecting the emotional well-being of this college population.

*“Yes, absolutely (speaking of her family’s prejudices about the subject of mental health), I buy and hide the medications that the psychiatrist prescribes for me.” (Lana, 22 years old)*

*“My dad is very much like, ‘No! You haven’t experienced anything’... that made me feel bad... when I told her that... I think she spoke to my psychologist and then she said: if this is going to make you feel better, that you can improve, it’s okay, I support you... but she is very different from my dad. (Pamela, 22 years old)*

Lana and Pamela’s stories show that fear of external judgment is not just an isolated emotion, but a manifestation of internalized stigma and family norms that minimize mental health. The need to hide psychiatric medication or to restrict who therapy can be discussed with reflects a climate of suspicion that contradicts the principles of containment and support necessary for any emotional care process. Combating external judgment not only involves working with those who seek help, but also with the environments that reproduce it.

The experiences described show that access to mental health care for LGB students cannot solely be understood as a matter of coverage or service availability, but as a phenomenon influenced by power relations, normative discourses, and climates of mistrust. The stigma surrounding therapy and psychiatry, experiences of discrimination in care settings, and the persistent fear of external judgment are concrete expressions of how inequality translates into psychological and social barriers that prevent timely emotional care. Including this dimension in the analysis not only enables one to situate the mental health needs of LGB students at the individual level, but also within a structural framework.

### Resignification of mental health as a right and self-care recognition of therapy as a valid tool

Recognizing therapy as a valid tool represents a turning point in the way LGB college students relate to their mental health. This subcategory takes on particular relevance in contexts where both sexual orientation and emotional distress have historically been stigmatized. As participants began to identify anxiety and depression as legitimate, common problems, they also become open to seeking professional support as part of their self-care.

*“I feel that the main ones, the ‘basics,’ so to speak, that need to be addressed would be anxiety and depression. Those are obviously the main ones, but it should be talked about more, because there is a different concept and people don’t know that help is available. Yes, there should be more information so that people are encouraged to go to therapy.” (Edna, 20 years old)*

*“I think that if you need help, accompaniment is excellent. Many times you need it and you don’t go, but taking care of your mental health is just as important as taking care of your physical health. I feel like we should all do it.” (Claudia, 22 years old)*

These testimonials show that when therapy is understood as a legitimate form of self-care, it expands the opportunity for LGB college students to access mental health services without feeling shame or guilt. Validating emotional experiences and recognizing that “feeling bad” does not imply weakness, but humanity, becomes a political act of affirmation and resistance to contexts that have historically pathologized both sexual diversity and psychological suffering.

#### Change in the family’s attitude to psychological care.

Exploring changes in family attitudes toward psychological care allows us to understand how immediate environments, particularly families, influence whether LGB university students seek or shun emotional support. In many contexts, mental health has been treated as a taboo subject, associated with severe conditions, abnormality, or weakness. However, when families begin to reframe therapy as a legitimate, everyday practice of self-care, stigma is reduced and it becomes easier for students to approach care without fear or guilt.

*“At first, I thought psychiatrists were only for people who were already very ill, because in my family, we only talked about them when someone was seriously ill. But I realized that it’s not like that, that you can go for many reasons. “...my mom also changed her attitude when she saw that in our family we started talking more about therapy…” She no longer sees it as something bad, but as something normal.” (Jazmín, 20 years old)*

Jazmín’s testimonial shows that when families begin to talk about mental health without prejudice, it creates new conditions for LGB students to access the help they need. This change not only reflects a generational shift, but also an opportunity: to transform the inherited narrative of suffering as shame into a vision of psychological care as part of dignified, humane care. Addressing this subcategory is essential if we seek to understand the real mental health needs of LGB university students, since it reveals the impact of family ties on access to, and the continuity and legitimacy of therapeutic support.

#### Integrating emotional well-being as part of personal development.

Recognizing mental health as part of personal development represents a crucial stage in understanding the needs of LGB college students. This subcategory makes it possible to see how young people, based on their experiences, begin to identify therapy not as an extreme or pathological resource, but as a tool for self-knowledge, emotional regulation, and human growth. This redefinition not only implies a break with traditional stigmas but is also an active appropriation of well-being as part of their life projects.

*“Well, at the beginning it was very difficult. I didn’t tell anyone, I felt so sorry. Now I feel that it says a lot about a person’s maturity to understand that it’s necessary to go to therapy, that sometimes you need to cry and find the tools to cope with difficult times. I used to think it was a place for crazy people, but now I see it’s for sane people... (Lana, 22 years old)*

*“I’ve heard things about those who are medicated like that, and I don’t know if it’s right, because there are things that can’t be resolved just by talking about them. It’s not just about what happened to you, there are also chemical imbalances. So I think psychiatry is fine, but I think there should also be psycho-humanistic support…” (Eloisa, 22 years old)*

What Lana and Eloísa mention illustrates a significant shift in the way young LGB people understand and value their mental health. By integrating it as a legitimate dimension of self- care, new ways of confronting discomfort emerge from a place of agency rather than shame. This finding is key to the objective of this research, as it shows that mental health needs are not only expressed in terms of illness, but also in the urgent need for care models that recognize emotional development as a right and a daily practice.

## Discussion

The objective of this study was to explore the mental health needs of LGB university students from an intersectional perspective, considering how sexual orientation intersects with structural factors such as gender, social class, family background, and academic status. The findings reveal that the process of exploring and accepting sexual orientation is deeply affected by structural stigma and unequal access to services and information about diversity. In line with the minority stress theory [8], the testimonials reflect a relationship between distal stress, derived from institutional invisibility and discrimination, and proximal stress, manifested in fear, doubt, guilt or emotional vigilance. These results are consistent with studies that have documented the negative effects of heteronormativity on the mental health of LGB youth [40,41], especially when sexual diversity intersects with precarious conditions, academic pressure, or the lack of support networks.

The absence of comprehensive sex education and the scarcity of positive representations of sexual diversity in educational settings create barriers to self-knowledge and self-acceptance. The testimonials of the participants show that uncertainty about their sexual identity is linked to the lack of accessible references, leading some to turn to cinema or other cultural spaces to understand their own feelings [35]. These findings confirm previous research that has identified the central role of information in identity formation and the importance of inclusive educational environments

The narratives also reveal the importance of heteronormativity in the construction of sexual orientation and its impact on the self-image of LGB people. Self-stigma manifests itself in feelings of guilt, shame, and the desire to fit into the heterosexual norm. In the case of Carmen and Pedro, their stories reflect the impact of the pathologization of sexual diversity on their emotional well-being, which is consistent with studies documenting the effects of minority stress on the mental health of LGB youth [39,41].

Testimonials about coming out in the family environment show a variety of responses, from overt rejection to subtle denial. In some cases, humor is used as an affect regulation tool to minimize the seriousness of the disclosure, constituting a form of invalidation of LGB identity. This phenomenon has previously been documented in research exploring family barriers to accepting sexual diversity [20]. Furthermore, the minority stress model suggests that ambiguous or rejecting responses influence identity construction and long-term mental health [8].

Beyond the experience of being LGB, students also face academic and economic stressors that impact their emotional well-being. The transition from virtual to in-person learning following the COVID-19 pandemic exacerbated challenges related to time management, academic load, and performance pressure. While these experiences are common to the general student population, LGB students face a double burden of stress when these challenges are combined with structural discrimination and a lack of support networks. Financial precariousness also appears as a determining factor in the university experience, not only affecting access to mental health services, but also emotional security and personal stability

The findings also reveal the existence of a “double closet,” where students not only conceal their sexual identity but also their need for psychological care for fear of social judgment. The pathologization of sexual diversity and the devaluation of emotional well-being mean that many people avoid seeking help, perpetuating cycles of discomfort and isolation. Moreover, the testimonials reveal structural barriers within the mental health system, including the lack of training of professionals in sexual and gender diversity, as well as the persistence of pathologizing approaches [42,43].

The findings of this study therefore show that the mental health needs of LGB university students cannot be understood in isolation or reduced to their sexual orientation. Rather, they emerge from the interaction between multiple dimensions of vulnerability and exclusion operating simultaneously: from family rejection and financial hardship to discrimination in health services and the emotional burden of concealing both sexual identity and psychological distress. The double closet phenomenon described by participants highlights how fear of social judgment remains a significant barrier to timely access to care [8,17]. Given this situation, university mental health policies should therefore integrate an intersectional perspective that not only considers access but also the quality, cultural relevance, and emotional safety of services. Ensuring the emotional well-being of LGB students involves transforming the structural conditions that perpetuate minority stress, as well as fostering stigma-free therapeutic spaces with professionals trained to provide support based on respect, affirmation, and understanding of diverse backgrounds.

## Conclusions

This qualitative study sought to explore the mental health needs of LGB college students from an intersectional perspective, recognizing how multiple social, structural, and identity factors impact their emotional well-being. The findings show that this population faces significant barriers to accessing, remaining in, and obtaining effective mental health care, due to the stigma surrounding sexual orientation and psychological distress. The concept of the “double closet” emerges as a key analytical tool for understanding how the need to conceal both identity and emotional suffering becomes an additional psychological burden violating the right to care.

The study also reveals that, while LGB students value the importance of psychological care, discriminatory practices and social representations persist that hinder their access to timely, quality services. This highlights the urgent need to strengthen training in cultural competency, intersectionality, and gender perspective among mental health care providers in universities.

In this respect, the results confirm that addressing the mental health needs of LGB university students requires a committed institutional response that recognizes the unique characteristics of their backgrounds and implements affirmative action to guarantee their right to well-being. As spaces for education and social transformation, public universities must take on the challenge of eradicating intersectional stigma through inclusive policies, psychosocial support programs, awareness campaigns, and safe educational spaces that encourage both the expression of diversity and the search for help without fear of judgment or exclusion.
